# Institutionalizing Telemedicine Applications: The Challenge of Legitimizing Decision-Making

**DOI:** 10.2196/jmir.1669

**Published:** 2011-09-28

**Authors:** Paolo Zanaboni, Emanuele Lettieri

**Affiliations:** ^1^Norwegian Centre for Integrated Care and TelemedicineUniversity Hospital of North NorwayTromsøNorway; ^2^Department of Management, Economics and Industrial EngineeringPolitecnico di MilanoMilanoItaly

**Keywords:** Telemedicine, decision making, institutionalization, technology assessment, implementation, program sustainability

## Abstract

During the last decades a variety of telemedicine applications have been trialed worldwide. However, telemedicine is still an example of major potential benefits that have not been fully attained. Health care regulators are still debating why institutionalizing telemedicine applications on a large scale has been so difficult and why health care professionals are often averse or indifferent to telemedicine applications, thus preventing them from becoming part of everyday clinical routines. We believe that the lack of consolidated procedures for supporting decision making by health care regulators is a major weakness.

We aim to further the current debate on how to legitimize decision making about the institutionalization of telemedicine applications on a large scale.

We discuss (1) three main requirements— rationality, fairness, and efficiency—that should underpin decision making so that the relevant stakeholders perceive them as being legitimate, and (2) the domains and criteria for comparing and assessing telemedicine applications—benefits and sustainability.

According to these requirements and criteria, we illustrate a possible reference process for legitimate decision making about which telemedicine applications to implement on a large scale. This process adopts the health care regulators’ perspective and is made up of 2 subsequent stages, in which a preliminary proposal and then a full proposal are reviewed.

## Introduction

Telemedicine is the use of communications networks for delivering health care services and medical education from one geographical location to another [1]. In this regard, telemedicine is generally acknowledged as a subset of eHealth applications, which are health care services and information delivered or enhanced through the Internet and related technologies [2].

Telemedicine is not new. Although a variety of telemedicine applications have been trialed worldwide over the last decades [3,4], telemedicine is still an example of major potential benefits that have not been fully attained [5]. Many applications that were adopted with great expectations quickly became failures and were subsequently abandoned [6]. Health care regulators are still debating why institutionalizing telemedicine applications on a large scale has been so difficult [5,7] and why health care professionals are often averse or indifferent to telemedicine applications, thus preventing them from becoming part of everyday clinical routines [8,9].

**Figure 1:**
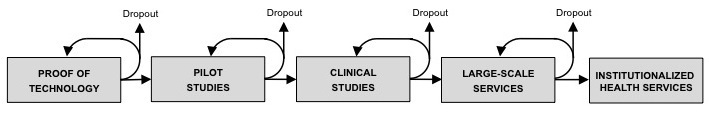
Implementation process of telemedicine applications.

We believe that the reasons are many, but among others the lack of consolidated procedures for supporting decision making is a major weakness [10-13]. Recently the discipline of health technology assessment (HTA) has been acknowledged as the gold standard for the assessment of health care technology [14]. However, the major emphasis is on drugs, equipment, and medical devices; thus, telemedicine applications are far from having a reference assessment procedure [6]. Scholars of technology assessment are still debating whether telemedicine applications can be included within the scope of HTA [7]. Other studies investigated the problems [10] and conditions for successful implementation of telemedicine applications, identifying the most critical organizational [6,15] and contextual factors [16]. Within this debate, the normalization process theory developed by May et al [17] offers a theoretical lens through which to understand the implementation, embedding, and integration of complex health care interventions into everyday practice. Normalization process theory is today a well-established theory applied to several applications, including telemedicine [7]. However, this theory is more descriptive than prescriptive, and thus more pragmatic procedures for supporting decision making with respect to large-scale institutionalization of telemedicine applications are still missing [18]. Institutionalization is the last phase within the reference pathway that telemedicine applications follow to enter into clinical routine (Figure 1). Decisions about the institutionalization of telemedicine applications are usually taken by health care regulators, governments, and authorities in countries with a national health care service, such as the United Kingdom, France, Italy, and Norway.

In this Viewpoint, we consider this perspective, since we believe that in these cases the institutional actors (ie, health care regulators, governments, and authorities) are required by relevant stakeholders (eg, health care providers, patient groups, scientific societies, insurance companies, and technology suppliers) to legitimate their decision making about whether to institutionalize new health care technologies, such as telemedicine.

In particular, decision making about which telemedicine applications should be prioritized for large-scale implementation is tremendously complex [19]. In this context, we believe that a major issue for both academicians and practitioners should be to discuss how this peculiar decision-making exercise should be organized and which criteria should be adopted to support the selection of the most promising telemedicine applications.

We aim to further the debate on how to legitimize decision making about telemedicine applications by adding new elements to this debate. In particular, we discuss (1) the main requirements that should underpin this exercise for being perceived as legitimate by relevant stakeholders, and (2) the domains and criteria that should be taken into account for comparing and assessing different applications. Finally, we illustrate a possible reference decision-making process for telemedicine applications that might be taken as basis for both practical applications and academic discussions, and possibly extended to other eHealth applications.

## Legitimizing Decision Making

**Figure 2:**
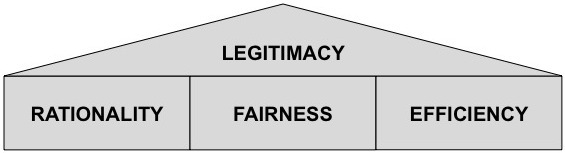
Requirements for legitimate decision making.

Health care is a complex ecosystem of stakeholders [20]. Decision making about which telemedicine applications should be implemented into a large scale affects a variety of stakeholders and whether their goals are met. In this view, it is critical that they perceive this exercise as legitimate [7]. Legitimacy refers to “a generalized perception or assumption that the actions of an entity are desirable, proper, or appropriate within some socially constructed system of norms, values, beliefs, and definitions” [21]. As a consequence, any organizational process should take into account the embracement of socially accepted techniques and procedures for being legitimate [22]. With respect to the peculiar case of the assessment of telemedicine applications, any recommendation should take into account a multiple-stakeholder perspective. Legitimization by the key stakeholders (ie, health care providers, patient groups, and technology suppliers) is gained when the decision-making exercise is respectful of their views and conforms to their perceptions of what is appropriate. Evidence from previous research suggests that 3 requirements are salient to gain stakeholders’ approval (Figure 2). First, decision making should be rational [23]. Second, decision making should be fair [24]. Third, decision making should be efficient [20]. In the following, we briefly discuss these requirements.

### Rationality

Rationality of decision making is generally addressed through the adoption of multicriteria approaches. Decision making that covers a variety of criteria takes explicit account of the multifaceted nature of decisions under discussion and thus supports decision makers in exploring what really matters to key stakeholders [25]. All relevant goals and assessment criteria should be adequately captured and made accountable for decision makers [26], since shortcomings might arise if these goals and criteria have not been clarified [27]. Preferences between options should refer to an explicit set of objectives [28]. In this view, the selection of those telemedicine applications that appear to be the most promising should explicitly take into account the relevant and often contrasting goals. The peculiar nature of telemedicine applications and their expected performance should be assessed against multicriteria frameworks that facilitate a synthesis—and thus a conciliation—of different domains and perspectives. Previous contributions suggested a variety of criteria [11,13,29,30]; we believe that these criteria should be grouped into 2 main domains: (1) benefits-related criteria; and (2) sustainability-related criteria [31]. The last group is particularly critical for us. With “sustainability” we refer to the capability of use of a health care service to become routine and deliver high-quality and efficient care over time. A list of criteria that may be adopted for both the domains is discussed below.

### Fairness

The second requirement for legitimacy is fairness. Decision making should be fair, accountable, and transparent [32]. This is particularly relevant when decision makers have contrasting views and goals [33]. The accountability for reasonableness (A4R) framework [32] offers a pragmatic solution to this issue, since it facilitates basing decisions on only those reasons that everybody will agree on and support [34]. The framework identifies 4 conditions that should be met by “fair-minded” decision makers: (1) relevance, (2) publicity, (3) revisability, and (4) enforcement [26]. Despite interest in the A4R framework, we must acknowledge that the literature offers little guidance on what a fair and legitimate process might look like in practice, and the process itself is still a “black box” [34]. The 4 conditions posited by the A4R framework are significant for the assessment of telemedicine applications. Relevance—required also for rationality—is necessary to address competing rationales and promote relevant stakeholders’ agreement. Publicity is critical for continuous learning. Proponents of telemedicine innovations must access, not just by demand, previous assessment exercises to learn about which criteria had been selected to compare benefits and sustainability in order to improve their further proposals. In particular, shortcomings of ongoing or concluded studies should be acknowledged and diffused to improve the reliability of further proposals [35]. In this view, adopting an appraisal-based process is also relevant for creating a trustworthy setting and facilitating learning and continuous improvement over time. Finally, decision makers must ensure that the above 3 conditions are met in order to win stakeholders’ legitimization.

### Efficiency

We believe that efficiency is another key requirement for legitimate decision making [20], which should be timely and cost contained. The literature does not completely clarify how an efficient decision-making exercise should be organized with respect to technology assessment. Despite this limitation, observation of current practices by highly regarded agencies seems to acknowledge that a 2-stage decision-making exercise should be used to pursue timely and cost-contained decisions. A first example is the call for proposals within the Seventh Framework Program (2007–2013) of the European Commission. Applicants are required first to submit a limited outline proposal. All successful proposals are then invited to submit a full proposal [36]. Narrowing the view to health care, the National Institute for Health and Clinical Excellence (NICE) in the United Kingdom adopted a similar process [37]. The process [38] is based on selection of new interventions first, followed by a structured appraisal of clinical effectiveness, cost effectiveness, and wider implications for the National Health Service as a whole. Emerging technologies that might require a NICE evaluation are identified by the National Horizon Scanning Centre at the University of Birmingham, England. First, a panel of experts prioritizes technologies for assessment. Second, the selected technologies are assessed and briefings are delivered to decision makers. Narrowing the view to telemedicine applications, the Scottish Telemedicine Initiative [39] invites organizations associated with the NHS in Scotland to submit assessment proposals to promote the implementation of telemedicine applications in Scotland. The procedure is also based on a 2-stage review process.

Based on these examples, we argue that decision-making exercise should possibly be organized through 2 sequential gates, with the first round aimed at selecting the most promising telemedicine applications and the second round aimed at selecting applications for large-scale implementation.

### Criteria for Decision Making

As mentioned, rational decision making requires a multicriteria assessment. A variety of prescriptive multicriteria frameworks [11,13,29,30] converge on the identification of a list of assessment dimensions for telemedicine applications that should be reviewed by decision makers. Key dimensions include technical feasibility, legal and ethical issues, clinical effectiveness, economics, equity of access, acceptance by providers and patients, and organizational impacts. As previously mentioned, we think that decision makers would receive better support if the list of criteria covering these dimensions were grouped into 2 main domains: benefits and sustainability [31].

In this regard, we analyzed the literature to collect past contributions about benefits and sustainability measures. Our analysis was not intended to provide a systematic review of assessment criteria. Rather, it aimed at offering a first draft of how these 2 domains might be assessed through specific criteria. In the peculiar case of telemedicine applications, 3 main benefits-related criteria (Table 1) may be recognized: (1) clinical outcomes, (2) cost containment, and (3) access.

**Table 1:** Benefits-related criteria

Dimension	Measures	References
Clinical outcomes	Diagnostic accuracy	13,40–42
	Appropriateness of clinical decisions	42–45
	Therapeutic efficacy/effectiveness	13,41,46
	Timeliness of care	13,30,47,48
	Mortality	13,42,49–51
	Morbidity	13,50,52
	Disease-related measures	11,13,29,30,42
	Quality of life	11,13,42
	Hospitalizations	42,49,53,54
	Length of hospital stay	42,51,53
	In-clinic visits	42,45,49
	Emergency department accesses	45,53,55
Cost containment	Cost of the service	29,30,42,56–58
	Relative efficiency	11,29,30,58–60
Access	Geographic accessibility	29,30,42,45,61
	Availability	13,45,61,62
	Waiting lists	13,63
	Affordability	45,64

Clinical outcomes [30] consist of a wide set of measures that determine the effects of the implementation of telemedicine applications on patients’ health status. They are diagnostic accuracy [13,40-42], appropriateness of clinical decisions [42-45], therapeutic efficacy/effectiveness [13,41,46], timeliness of care [13,30,47,48], mortality [13,42,49-51], morbidity [13,50,52], other efficacy/effectiveness or disease-related measures [11,13,29,30,42], quality of life [11,13,42], hospitalizations [42,49,53,54], length of hospital stay [42,51,53], in-clinic visits [42,45,49], and emergency department accesses [45,53,55].

Cost containment is intended to measure the value of resource use associated with an intervention [29], thus allowing an understanding of whether a telemedicine application is cost saving or cost effective. First, the cost of a telemedicine application has to be measured and compared with the alternative clinical practice [29,30,42,56-58]. Second, relative efficiency with other alternative health care strategies is critical for decision making when resources are limited [11,29,30,58-60].

Telemedicine applications are claimed to improve access to health care services, especially for people living in rural or remote areas where medical professionals and facilities are scarce or absent [29]. Access includes a set of specific dimensions describing the fit between the patient and the system, including geographical accessibility of patients [29,30,42,45,61], availability of health care resources and professionals [13,45,61,62], waiting lists for secondary care [13,63], and affordability of health care services [45,64].

A growing body of evidence suggests that decision makers should also take into account sustainability-related criteria when assessing health care technologies [26,31]. As mentioned, we refer to sustainability as the capability of a telemedicine application to deliver high-quality and efficient care over time. In this respect, sustainability is not a single concept, but is an umbrella of different dimensions. Five main dimensions (Table 2) may be recognized. They are (1) technological sustainability, (2) clinical sustainability, (3) organizational sustainability, (4) economic sustainability, and (5) contextual sustainability.

**Table 2:** Sustainability-related criteria

Dimension	Measures	References
Technological sustainability	Integration and interoperability	11,65,66
	Use over an extended period of time (proof of time)	30,58
Clinical sustainability	Malpractice, adverse events, and uncertainty in clinical practice	29,67,68
Organizational sustainability	Acceptance and satisfaction	13,29,30,42,69
	Staff, skills, learning, and training	30,70–73
	Leadership, communication, roles, and responsibilities	71–73
	Changes in organizational structure and work processes	42,70,72,73
	Collaboration, cooperation, partnership, and networks	71–73
Economic sustainability	Cost structure	11,13,42,56–58,60
	Total investment	13,57,58
	Level of use	13,56–58
	Costs for patients and caregivers	13,42,57–59
Contextual sustainability	Respect of ethical requirements	67,68
	Respect of legal requirements	30,67,68,74

The technological sustainability of a telemedicine application specifically addresses its integration and interoperability [11,65,66] with the existing technologies in a health care organization. Additionally, the telemedicine application has to be used over an extended period of time [30,58], thus attesting to the so-called *proof of time*.

Clinical sustainability addresses whether the clinical benefits will be maintained over time. Malpractice, including adverse events and uncertainty in clinical practice, should therefore be carefully considered [29,67,68].

Organizational sustainability refers to the readiness of organizations where the telemedicine application is implemented and potential resistance to change. The successful adoption of telemedicine applications depends on the acceptance and satisfaction of health care professionals, patients, caregivers, and other users [13,29,30,42,69]. Internal resistance to change can be also driven by issues related to staff, skills, and learning and training needs [30,70-73]. Leadership, roles, and responsibilities [71-73] have to be carefully defined and communicated among the health care professionals while introducing a new technology. Additionally, telemedicine applications imply the introduction of changes within the organizational structure and work processes [42,70,72,73]. Collaboration, cooperation, partnership, and networks [71-73] are also required beyond the borders of the organization, in relation to other health care providers and stakeholders.

Economic sustainability refers to the economic value generation derived from the use of telemedicine applications and the capability to maintain it over time. Economic sustainability is related to the cost structure [11,13,42,56-58,60] and to the total amount of capital investments [13,57,58]. Moreover, the economic impact depends on the level of use of the telemedicine application [13,56-58] and on the costs sustained by patients and caregivers [13,42,57-59].

Finally, contextual sustainability is necessary for the long-term use of a service. Contextual sustainability of telemedicine applications refers to the respect of ethical [67,68] and legal requirements [30,67,68,74].

We believe that clustering criteria in terms of benefits and sustainability offers better support to decision makers, since this approach makes clear to all key stakeholders both the potential value of a telemedicine application and the difficulties in pursuing high performance over time. Rationality and accountability of decision making will benefit from the transparent disclosure of decision makers’ expectations in terms of benefits and sustainability, and this will facilitates stakeholders’ acceptance of the final decision.

## A Possible Reference Process for Decision Making

In this section, we illustrate a possible reference process for legitimate decision making about which telemedicine applications to implement on a large scale. As mentioned, we adopt the health care regulators’ perspective and we address the need for key stakeholders, such as health care providers, patient groups, and technology suppliers, to legitimize this process. Our proposal of a reference process is based on the previous discussion. In this regard, we argue for a process that is made up of 2 subsequent stages, in which a preliminary proposal and then a full proposal are reviewed: this structure improves efficiency and leverages on the real examples that we have previously illustrated.

### Stage 1: Assessment of Preliminary Proposals

The process would start with a request by an accredited subject (eg, hospitals, research centers) to decision makers to implement a large-scale telemedicine application. We assume a bottom-up process (and not top-down), since this perspective coheres to what happens in those countries that have a national health care system. The request derives from previous studies that enrolled a limited number of patients and providers, whose results justify the request to move to a larger scale. Subjects that were involved in these studies would submit a preliminary proposal.

The preliminary proposal is a document that provides decision makers with summary information about the telemedicine application under discussion. At this stage, all the preliminary proposals should be prioritized. Only those proposals with a satisfactory relevance score would be asked to submit the full proposal. This prioritization exercise would be conducted by an institutional committee (eg, members of governments or authorities) on the basis of the documentation provided. The possibility to establish an advisory panel as suggested in previous research [26] should be discussed. Preliminary proposals structure information in 2 main sections: (1) a description of the context into which the telemedicine application should be introduced, and (2) a description of the proposed telemedicine application and its benefits.

The description of the context (Table 3) provides information necessary to understand the nature of the problem that the telemedicine application aims to solve. For instance, a telemedicine application might address a clinical problem (eg, patients’ health conditions affected by a chronic disease), an economic problem (eg, the containment of present expenditure for a specific health need), or a problem of accessibility to health care services (eg, the provision of secondary care to rural areas). Published data about the problem under consideration should support decision makers in understanding its relevance at both the national and the local level in order to contextualize the assessment exercise. Moreover, a telemedicine application is often introduced as an alternative to current clinical practice, commonly face to face [75]. In these cases, decision makers expect that the telemedicine application will produce at least the same clinical outcomes as the conventional practice before being adopted into routine [11]. Telemedicine applications could be either supplementary [45], offering additional activities, or alternative, changing the way of providing a service [76]. More rarely, they address needs still uncovered by traditional practice, thus allowing access to services not otherwise available [77].

**Table 3:** Preliminary proposal: description of the context

Dimension	Information
Nature of the problem	Description of the problem (clinical, economic, access) the telemedicine application aims to solve
	Description of data that support the relevance of the problem at the national or regional level
Current clinical practice	Description of current clinical practice (if existing) to which the telemedicine application is presented as an alternative practice (integrative or substitutive)

The description of the proposed telemedicine application (Table 4) aims to provide that information necessary to prioritize the implementation on a large scale. First, the proposal must clarify the type of service (eg, doctor-to-doctor consultation via telemedicine systems [78]), the description of the target patients (eg, patients affected by a specific chronic disease), the technology used for providing the service (eg, Internet), and the subjects who will be involved or interested in the service provision. Second, the proposal must clarify the benefits expected from the implementation of a large-scale telemedicine application. These can be measured through clinical outcomes, costs, and access.

**Table 4:** Preliminary proposal: description of the proposed telemedicine application

Dimension	Information
Description of the service	Characterization of the type of telemedicine application
	Characterization of patients
	Description of technologies used to provide the service
	Description of subjects involved in the service and their roles
Expected benefits	Impact on clinical outcomes
	Impact on costs
	Impact on access

The institutional committee, after having examined all the information provided, would make a decision based on the content of the proposal and its relevance to the health care system. Three different replies might arise: (1) the preliminary proposal is accepted due to positive feedback, (2) the preliminary proposal is accepted with reservation due to satisfactory feedback with some weaknesses to be addressed, (3) the preliminary proposal is rejected due to unsatisfactory feedback. Proposals in the first or second scenarios would be asked to be completed as a full proposal.

### Stage 2: Assessment of Full Proposals

The full proposal provides detailed information about the telemedicine application. Full proposals would be assessed mainly by a board of experts, selected from a list of experts from different scientific fields (eg, medicine, sociology, health economics) who would be required to disclose their possible conflicts of interest. This board would produce recommendations for the institutional committee, responsible for the final selection.

The information collected into the full proposals is structured in 4 parts: (1) a description of the context in which the telemedicine application should be introduced, (2) a description of the proposed telemedicine application and its benefits, (3) documentation supporting the assessment, and (4) an assessment of the scientific basis and available evidence.

The context and the proposed telemedicine application in the full proposal contain the same information as in the preliminary proposal, but in more detail. Additional fields should require the specification of inclusion and exclusion criteria of enrollment, clinical protocol, technical requirements, expected and proper level of utilization, data and information exchanged, and methods for data collection and assessment.

The third part collects evidence that supports previous claims and figures. Studies, papers, and reports about the specific telemedicine application under consideration should be provided. The board of experts should verify the reliability of this evidence in terms of levels of evidence and strength of the recommendations [79].

The process would end with an assessment of the scientific basis. Both benefits-related criteria and sustainability-related criteria should be addressed as previously described in Table 1 and Table 2. Where information is available, the proponent should clarify the metrics used to measure the specific criteria, results, and references. The benefits-related criteria should be measured preferably with respect to a comparator (eg, control group of patients). Referring to the sustainability-related criteria, results and references should be classified with respect to the main stakeholders: patients and caregivers, health care professionals, hospitals, vendors and service centers, or the health care system.

At the end of the process, the board of expert should examine the context and the proposed telemedicine application and assess its benefits and sustainability with respect to a large-scale implementation. The institutional committee would then make the final decision. The implementation of a large-scale telemedicine application should finally require periodic monitoring to verify the correct implementation and level of use by health care providers, and the achievement of the expected benefits and its sustainability over time, and eventually to modify the clinical protocol or to interrupt the service.

## Conclusions

### Final Remarks

This Viewpoint provides new insights into the ongoing debate about the institutionalization of telemedicine applications. In particular, our discussion originates from the acknowledgement that a major weakness inhibiting the large-scale implementation of telemedicine applications is the lack of a legitimate reference process for decision making by health care regulators. Moving from this, we shed some light on (1) 3 main requirements that should characterize this process—that is, rationality, fairness, and efficiency, (2) some criteria for measuring the benefits and sustainability of a telemedicine application, and (3) what a legitimate process might look like in practice. We believe that these contributions will promote further discussion among practitioners and academicians about supporting the institutionalization of telemedicine applications.

### Practical Implications

Two examples from real-world practice may illustrate some implications of this discussion. First, health care regulators complain that HTA reports often do not provide them with all the information they require for decision making, since salient information such as organizational impacts and resistance to change are not fully disclosed [80]. This limitation is particularly true for telemedicine applications [81], since contributions in the literature are more interested in discussing clinical outcomes (in particular they aim to verify whether telemedicine services deliver at least the same effectiveness as current face-to-face ones) or technical feasibility (to persuade professionals that telemedicine services are safe and robust to possible technical failures). Our view is that decision making should be rational and accountable, and thus our examples of a preliminary proposal and a full proposal help to frame this requirement in 2 groups of criteria. By knowing which information is salient for supporting decision making, both the agencies that deliver HTA reports and scholars who publish the main results of their studies about new telemedicine applications will be able to increase the impact of their contributions. In fact, they will know in advance which information will be searched through their contributions.

Second, professionals (physicians in particular) are largely involved in clinical studies aimed at understanding the feasibility and the performance of telemedicine applications. This Viewpoint paper offers them some insights into how health care regulators should assess the possibility of implementing their innovations on a large scale. These studies often collect only a small part of the information that decision makers would require to make a decision. This would be a lost opportunity for both patients and professionals, and missing information will be collected through further studies. Consider a hospital involved by a technology supplier in a randomized controlled trial on telemonitoring of patients with chronic disease (eg, chronic heart failure, chronic obstructive pulmonary disease). By knowing in advance which information is salient for decision makers to implement this application on a larger scale, both physicians and technicians will be able to define the most appropriate study design and to collect all relevant data, such as professionals’ perception of utility and ease of use as predictor of potential resistance to changing current practice.

### Limitations

There are 2 main limitations to our proposed reference process for decision making. These limitations suggest avenues for future research and debates. First, we did not systematically review the literature to identify which criteria should be included in the proposed framework. As mentioned, our literature analysis was intended to provide a first example without attempting to be definitive.

Second, we adopted the health care regulators’ perspective for the proposed 2-stage process. As a consequence, it has been designed to support primarily governments and authorities in prioritizing which telemedicine applications should be institutionalized, and therefore funded, at a large scale. Although we believe that this perspective is the most relevant in countries that have a national health care system, such as the United Kingdom, France, Italy, and Norway, we acknowledge that other relevant insights could be gathered by adopting other perspectives, such as that of technology suppliers or insurance companies.

## Abbreviations

A4R: accountability for reasonableness
HTA: health technology assessment
NICE: National Institute for Health and Clinical Excellence
